# Enhanced Solubility and Biological Activity of Dexibuprofen-Loaded Silica-Based Ternary Solid Dispersions

**DOI:** 10.3390/pharmaceutics15020399

**Published:** 2023-01-24

**Authors:** Muhammad Asim, Marriam Nazir, Zunera Chauhdary, Muhammad Irfan, Syed Haroon Khalid, Sajid Asghar, Usra, Raed I. Felimban, Mohammed A Majrashi, Mohannad S. Hazzazi, Mohammed Alissa, Safa H Qahl, Ghulam Hussain, Azhar Rasul, Shahzad Ali Shahid Chatha, Ikram Ullah Khan

**Affiliations:** 1 Department of Pharmaceutics, Faculty of Pharmaceutical Sciences, Government College University Faisalabad, Faisalabad 38000, Pakistan; 2 Department of Medicine, Faisalabad Medical University, Allied Hospital, Faisalabad 38000, Pakistan; 3 Department of Pharmacology, Faculty of Pharmaceutical Sciences, Government College University Faisalabad, Faisalabad 38000, Pakistan; 4 Department of Physiology, Government College University Faisalabad, Faisalabad 38000, Pakistan; 5 Department of Medical Laboratory Technology, Faculty of Applied Medical Sciences, King Abdulaziz University, Jeddah 21589, Saudi Arabia; 6 Center of Innovation in Personalized Medicine (CIPM), 3D Bioprinting Unit, King Abdulaziz University, Jeddah 21589, Saudi Arabia; 7 Department of Pharmacology, College of Medicine, University of Jeddah, Jeddah 23890, Saudi Arabia; 8 Department of Medical Laboratory Sciences, Faculty of Applied Medical Sciences, King Abdulaziz University, Jeddah 22254, Saudi Arabia; 9 Hematology Research Unit, King Fahd Medical Research Center, King Abdulaziz University, Jeddah 22254, Saudi Arabia; 10 Department of Medical Laboratory Sciences, College of Applied Medical Sciences, Prince Sattam bin Abdulaziz University, Al-Kharj 11942, Saudi Arabia; 11 Department of Biology, College of Science, University of Jeddah, Jeddah 21589, Saudi Arabia; 12 Department of Zoology, Government College University Faisalabad, Faisalabad 38000, Pakistan; 13 Department of Chemistry, Government College University Faisalabad, Faisalabad 38000, Pakistan

**Keywords:** dexibuprofen, solid dispersions, Syloid 244FP^®^, Gelucire 48/16^®^ and Poloxamer 188^®^

## Abstract

The current study was designed to formulate ternary solid dispersions (TSDs) of dexibuprofen (Dex) by solvent evaporation to augment the solubility and dissolution profile, in turn providing gastric protection and effective anti-inflammatory activity. Initially, nine formulations (S1 to S9) of binary solid dispersions (BSDs) were developed. Formulation S1 comprising a 1:1 weight ratio of Dex and Syloid 244FP^®^ was chosen as the optimum BSD formulation due to its better solubility profile. Afterward, 20 TSD formulations were developed using the optimum BSD. The formulation containing Syloid 244FP^®^ with 40% Gelucire 48/16^®^ (S18) and Poloxamer 188^®^ (S23) successfully enhanced the solubility by 28.23 and 38.02 times, respectively, in pH 6.8, while dissolution was increased by 1.99- and 2.01-fold during the first 5 min as compared to pure drug. The in vivo gastroprotective study in rats suggested that the average gastric lesion index was in the order of pure Dex (8.33 ± 2.02) > S1 (7 ± 1.32) > S18 (2.17 ± 1.61) > S23 (1.83 ± 1.04) > control (0). The in vivo anti-inflammatory study in rats revealed that the percentage inhibition of swelling was in the order of S23 (71.47 ± 2.16) > S18 (64.8 ± 3.79) > S1 (54.14 ± 6.78) > pure drug (18.43 ± 2.21) > control (1.18 ± 0.64) after 6 h. ELISA results further confirmed the anti-inflammatory potential of the developed formulation, where low levels of IL-6 and TNF alpha were reported for animals treated with S23. Therefore, S23 could be considered an effective formulation that not only enhanced the solubility and bioavailability but also reduced the gastric irritation of Dex.

## 1. Introduction

Dexibuprofen (Dex) is the pharmacologically active enantiomer of racemic ibuprofen, differing in physicochemical properties. Like ibuprofen, Dex acts by inhibiting prostaglandin synthesis [1]. It is frequently used for relief of osteoarthritis, primary dysmenorrhea, musculoskeletal pain, and toothache [2]. Dex shows a better pharmacological activity, tolerability, and safety profile when compared with ibuprofen. However, it belongs to class II of the biopharmaceutical classification system (BCS) having low solubility (0.0684 mg/mL) and high permeability [3]. Thus, it shows variable bioavailability and causes difficulties in formulating oral and injectable products [4]. Therefore, it is imperative to improve the aqueous solubility and bioavailability of the Dex to gain maximum benefits. Several methods have been tested to enhance the aqueous solubility of hydrophobic active pharmaceutical ingredients (APIs), including salt formation, complexation, prodrug formation, particle size reduction, micelles, nanosuspensions, pH modification, and solid dispersion (SD) [5]. SD is considered to be a successful strategy to improve the dissolution profile of poorly water-soluble drugs by improving wettability. SD refers to the dispersion of one or more APIs in an inert carrier [6]. According to the number of carriers, there are two types of SDs, i.e., binary solid dispersion (BSD) and ternary solid dispersion (TSD). BSDs are composed of the drug and one carrier, while TSDs are composed of two carriers. In comparison, TSDs exhibit enhanced solubility, release, bioavailability, and stability of poorly water-soluble drugs [7].

Both hydrophilic and hydrophobic carriers are employed to establish SDs for improving the dissolution rate of the hydrophobic drug. The recent literature has revealed the importance of silica and its derivatives for the improvement of drug solubility and bioavailability. Mesoporous silica represents a porous carrier where the pore size ranges from 2 to 50 nm; it is available in ordered (MCM-41 and SBA-15) and nonordered form (Syloid 244FP^®^, XDP3150^®^, and AL1FP^®^) [8]. Some of the popular drug loading techniques for mesoporous silica carriers include filtration, melting, and solvent evaporation [9].

The amorphous API in a solid dispersion is thermodynamically unstable and may recrystallize to the more stable crystalline structure during dissolution or the storage phase, thereby decreasing solubility. To overcome these drawbacks, there is a need to establish the SD with the highest dissolution rate, prevent precipitation under supersaturation, inhibit recrystallization, and improve the physical and chemical stability of water-insoluble drugs using surfactants [10]. Poloxamers are polyoxyethylene–polypropylene block copolymer nonionic surfactants that are commonly employed as wetting agents, solubilizing agents, and surface adsorption excipients [11]. Poloxamers have demonstrated higher solubility enhancement when compared to polymers such as PEGs or complex-forming agents such as cyclodextrins [12]. Poloxamer 188^®^ is generally chosen for SDs owing to its low melting point (about 56–57 °C), surfactant properties, and oral safety. Gelucires are lipid-based carriers with self-emulsifying properties and are widely employed in pharmaceutical preparations for the solubility enhancement of poorly water-soluble drugs. Gelucires are mixtures of mono-, di-, and triglycerides with PEG esters of fatty acids [13]. The polyvinyl caprolactam–polyvinyl acetate–polyethylene glycol graft copolymer (Soluplus^®^) shows amphiphilic properties and is widely used for the development of solid solutions [14].

Hence, the objectives of this research work were to prepare BSDs and TSDs via the solvent evaporation method to increase the solubility, dissolution, and bioavailability of Dex. Binary complexes of drug and silica particles were designed to compare the effectiveness of nonordered mesoporous silica (Syloid 244FP^®^, AL1FP^®^, and XDP3150^®^). Gelucire 44/14^®^, Gelucire 48/16^®^, Poloxamer 188^®^, and Soluplus^®^ were used as ternary carriers for the development of TSDs. Optimized formulations were evaluated for drug–polymer interactions, morphology, and formulation stability. The in vivo evaluation investigated the gastroprotective effect, anti-inflammatory activity, and measurement of inflammatory markers such as TNF alpha and IL-6 in serum.

## 2. Materials and Methods

### 2.1. Materials

Dexibuprofen was gifted by Global Pharmaceuticals, Islamabad Pakistan. Gelucire 44/14^®^ and Gelucire 48/16^®^ were received as gift samples from Gattefossè, France. Poloxamer 188^®^ was procured from Sigma Aldrich. Soluplus^®^ was kindly provided by BASF (Ludwigshafen, Germany). Grace Discovery Sciences (United States) generously provided Syloid 244FP^®^, Syloid XDP3150^®^, and Syloid AL1FP^®^. Monobasic potassium phosphate was purchased from Daejung Reagents Chemicals and Metals Co., Ltd., Korea. Methanol, sodium hydroxide, and dichloromethane were acquired from Sigma Aldrich. Hydrochloric acid was procured from Lab-Scan analytical sciences.

### 2.2. Preparation of SDs

Initially, nine BSDs were developed using the solvent evaporation technique (Table 1). Briefly, Dex was dissolved in dichloromethane (DCM). This solution of drug was taken in a glass mortar, and the appropriate amount of Syloid^®^ was added while stirring at 300 rpm for 30 min. Later, this suspension was dried overnight in an oven at 38 °C. Dried SDs were scratched, passed through sieve number 40, and stored in airtight glass vials. All vials were placed in a desiccator until further use [15].

In the case of TSDs, 20 (Table 2) formulations were developed using the solvent evaporation technique. Initially, Dex was dissolved in DCM. Afterward, the second carrier (Gelucire 44/14^®^, Gelucire 48/16^®^, Poloxamer 188^®^, or Soluplus^®^) was added in different percentages. When the second carrier was completely dissolved, optimized nonordered mesoporous silica was added. After complete drying, ternary complexes were kept in closed glass vials in a desiccator until further analysis.

### 2.3. Preformulation Studies

In preformulation studies, the BSDs of Dex were evaluated for solubility enhancement. The solubility of each formulation was checked in distilled water, pH 1.2, and pH 6.8, and then assessed for an increase in solubility in comparison to pure Dex. Afterward, the TSDs of Dex were developed with Gelucire 44/14^®^, Gelucire 48/16^®^, Poloxamer 188^®^, and Soluplus^®^. Lastly, their solubilities were tested in distilled water, pH 1.2, and pH 6.8.

### 2.4. Analysis of Drug Content

SDs equivalent to 10 mg of Dex were dissolved in 10 mL of methanol in a volumetric flask and vortexed. Then, 1 mL of sample was taken and diluted with methanol and assayed using a spectrophotometer (CECIL 7400-S, Cambridge, England) at 222.5 nm. Absorbance values were used to determine the drug content with a previously constructed calibration curve [16].

% drug content = actual amount of drug calculated/amount of drug initially added × 100.

### 2.5. Solubility Studies

Solubility studies were performed to estimate any increase in the solubility of Dex. In a falcon tube, 3 mL of solvent (1.2 pH buffer, 6.8 pH buffer, or distilled water) was added. Dex or SDs were introduced pinch by pinch in the respective solvent and vortexed to get a supersaturated solution. Falcon tubes were kept in a water bath shaker at 37 °C and shaken at 75 rpm for 72 h to reach equilibrium. After 3 days, falcon tubes were removed and centrifuged at 6000 rpm for 30 min. The supernatant was collected and passed through a 0.45 µm nylon syringe filter. The filtrate was appropriately diluted and assayed spectrophotometrically at 222.5 nm to calculate the drug content. Each measurement was run in triplicate [17].

### 2.6. Practical Yield

The percentage yield gives clue about the efficiency of the process. It is a ratio of the real output to theoretical output, as given in the equation below [18].

Percentage yield = actual yield/theoretical yield × 100.

### 2.7. Solid-State Characterization

#### 2.7.1. Fourier-Transformed Infrared Spectroscopy (FTIR)

FTIR was used to identify functional groups and assess the drug–excipient interactions. FTIR spectra of Dex and the optimized formulations (S1, S18, and S23) were obtained by scanning (BRUKER Tensor II-Alpha, Berlin, Germany) in the range of 4000–500 cm^−1^ at step length of 2 cm^−1^ in transmittance mode [19].

#### 2.7.2. X-ray Diffraction (XRD)

The crystalline or amorphous nature of pure Dex and optimized SDs (S1, S18 and S23) was studied using the powder X-ray diffraction technique. The X-ray diffractometer (Tongda TD-3500) was operated over the 2θ range of 10°–40°, at a speed of 3°/min, current of 2.5–30 mA, and voltage of 20–40 kV [20].

#### 2.7.3. Surface Morphology

The surface morphology of pure drug and Dex-loaded SDs (S1, S18, and S23) was examined at different resolutions using scanning electron microscopy (SEM) at 2 keV and 9 mm working distance. Each sample was placed on an aluminum stub with the help of double adhesive tape and sputter-coated with gold [21].

#### 2.7.4. Thermal Analysis

Differential scanning calorimetry (DSC) and thermogravimetric analysis (TGA) curves were concurrently obtained on an SDT Q 600 TA Universal instrument with 3–5 mg of sample. The pure drug and optimized formulations (S1, S18, and S23) were heated from 20 to 400 °C under nitrogen flow at a heating rate of 10 °C/min [22].

### 2.8. In Vitro Dissolution Studies

The drug release of Dex and optimum SDs was estimated by carrying out an in vitro dissolution test. Pure Dex and selected formulations containing 20 mg of drug were placed in 500 mL of dissolution medium (pH 1.2 or pH 6.8). The USP Apparatus II (PTWS 3CE, Pharma test, Hamburg, Germany) was set at 37 °C and 100rpm. Aliquots of 5 mL for each formulation were taken and replenished with 5 mL of fresh medium to maintain sink conditions. Samples were filtered through a 0.45 μm nylon syringe filter and assayed for Dex content at 222.5 nm using a UV/visible spectrophotometer (CECIL 7400-S, Cambridge, UK). All experiments were performed in triplicate.

### 2.9. Properties of the Powder

#### Density and Flowability

A graduated cylinder of 25 mL was used to determine the bulk and tapped densities of optimum formulations. After weighing 1 g of given sample, it was gently transferred to the graduated cylinder. The value of bulk density was calculated from the ratio of mass of powder and read volume [23]. For tapped density, the powder was tapped 100 times to allow the powder volume to achieve a plateau [24]. Three parameters are generally used to determine the flow properties of powder, i.e., Carr’s index, the Hausner ratio, and the angle of repose [25]. Carr’s index and the Hausner ratio were determined as follows:

Carr’s index = (tapped density − bulk density)/tapped density × 100.

Hausner ratio = tapped density/bulk density.

The funnel method was adopted to calculate the angle of repose. The radius (r) and the height (h) of the pile of powder were measured to determine the angle of repose as follows:

Angle of repose (θ) = Tan^−1^ [height (h)/radius (r)].

### 2.10. Estimation of Gastroprotective Effect

Albino rats weighing 160–240 g of either sex were selected to compare the ulcerogenic potential of Dex and optimized formulations. Animals were divided into five groups. Animals were kept at a temperature of 25 ± 3 °C under a 12 h light/dark cycle for 14 days prior to the experiment. The Ethical Review Board of Government College University Faisalabad approved all the protocols via letter with reference no. GCUF/ERC/2198. The animals fasted for 12 h before commencement of the experiment but were allowed to drink water freely. S1, S18, S23, and pure Dex were given orally (120 mg/kg) for 5 days in a suspension of carboxymethyl cellulose (CMC) (1 mL of 0.5% *w*/*v*). All animals in the control group only received 1 mL of suspension (0.5% *w*/*v* CMC). After 5 days, animals were sacrificed after 4 h of dosing [26].

#### 2.10.1. Macroscopic Scoring

Isolated stomachs were cleaned in isotonic saline solution after opening them along the greatest curvature. A magnifying glass was used to examine the mucosal damage. Ulcers were measured using a digital Vernier caliper. An arbitrary score (AS) was given to ulcers: “0” for no lesion or ulcer, “0.5” for one or more ulcers with length less than 1 mm, “1” for ulcers/lesions with length 1–2 mm, and “2” for ulcers/lesions with length >2 mm. The gastric lesion index (GLI) was calculated as follows [27]:

Gastric lesion index = AS × No. of lesions.

#### 2.10.2. Microscopic Analysis

For histopathological examination, stomachs were preserved in 20% *v/v* formalin buffer solution. Hematoxylin and eosin (H&E)-stained slides were observed under an Accu-scope 3000-LED microscope fitted with a 5 MP camera.

### 2.11. Anti-Inflammatory Effects

The anti-inflammatory effects of pure Dex and optimized formulations were determined using carrageenan-induced hind paw edema in albino rats weighing 140–160 g of either sex. Initially, the thickness of the right hind paw of each rat was measured using a digital Vernier caliper. Pure Dex and optimized formulations were given orally to the test groups at a dose of 12 mg/kg in 0.5% *w*/*v* CMC. Only 1 mL of 0.5% *w*/*v* CMC was administered orally to the control group. Then, 30 min after the administration of doses, 0.1 mL of 1% (*w*/*v*) freshly prepared carrageenan suspension in normal saline was injected into the control group and test groups to induce acute edema in the plantar region of the right hind paw. The increase in paw volume was measured at 60, 120, 180, 240, 300, and 360 min after carrageenan injection [26]. The percentage anti-inflammatory activity (AA) was determined using the following formula [28]:

AA (%) = (Ct − Co) control − (Ct − Co) treated/(Ct − Co) control × 100,(1)

where Ct is the right hind paw thickness (mm) at time “t”, Co is the right hind paw thickness (mm) before carrageenan injection, (Ct − Co) Control is the increase in paw size after carrageenan injection into control rats at time “t”, and (Ct − Co) Treated is the increase in paw size after carrageenan injection into treated rats at time “t”.

#### 2.11.1. Serum TNF Alpha and IL-6

After sacrificing rats, blood was collected in a falcon tube and kept for 15 min, before centrifuging at 6000 rpm for 20 min. The serum was collected in Eppendorf tubes and stored in a freezer until further use. The serum levels of TNF alpha and IL-6 were estimated using ELISA kits in compliance with the manufacturer’s instructions [29].

#### 2.11.2. Histopathological Analysis of Paw

The carrageenan-induced rat’s paws were dissected and preserved in 20% *v/v* formalin buffer solution. Sections were prepared as described earlier, and histopathological variations were observed under an Accu-scope 3000-LED microscope.

## 3. Results and discussion

### 3.1. Preformulation of SD

#### 3.1.1. Evaluation and Optimization of BSDs

Dex is a BCS class II drug with aqueous solubility of 0.0684 mg/mL. Therefore, it is imperative to enhance its solubility using an appropriate technique to achieve maximum therapeutic benefits. For this purpose, BSDs of Dex were prepared using Syloid 244FP^®^, XDP3150^®^, and Al1FP^®^ with different characteristics, as outlined in Table 3. These nonordered mesoporous silica carriers were selected due to the presence of a siloxane group with an oxygen–silicon backbone (–Si–O–Si–) and three types of silanol groups (≡Si–OH) that act as adsorption site. Furthermore, these porous silica materials can enhance the solubility of APIs by inhibiting crystallization through physisorption and capillary action [30].

##### Drug Content, Solubility, and Percentage Yield of BSDs

Initially, three different syloid grades were assessed in different ratios of drug to syloid, i.e., 1:1, 1:2, and 1:4, for solubility enhancement, as shown in Table 4. Among all BSD formulations, S1 containing a 1:1 weight ratio of Dex and Syloid 244FP^®^ presented the best drug entrapment (97%) and highest solubility of drug in distilled water (6.21 times), pH 1.2 (1.75 times), and pH 6.8 (24 times), when compared to pure drug (Table 4). This may be due to the attributes of Syloid 244FP^®^ (Table 3), whereby drug molecules are better confined to the mesoporous pores. The yield of all formulations ranged between 83% and 96%, verifying the efficiency of the solvent evaporation method for the preparation of BSDs. Therefore, S1 containing 1:1 of Dex and Syloid 244FP^®^ was selected as a suitable formulation for the development of TSDs owing to its better solubility, drug content, and practical yield.

#### 3.1.2. Evaluation and Optimization of TSDs

Although BSDs are able to enhance the dissolution profile, the supersaturation of APIs can lead to the precipitation of drug and a reduction in its concentration. Consequently, its bioavailability may decrease. Furthermore, during manufacturing and storage, the drug may transform into a crystalline or amorphous state [31]. In order to overcome these problems and bypass the gastric side-effects of the drug, TSDs were developed.

##### Drug content, Solubility, and Percentage Yield of TSDs

TSDs were prepared using two lipophilic carriers (Gelucire 44/14^®^ and Gelucire 48/16^®^) and two hydrophilic carriers (Poloxamer 188^®^ and Soluplus^®^) at different concentrations, as mentioned in Table 2. It was observed that free-flowing dispersions were obtained with lipid carriers up to 40%, beyond which a sticky and agglomerated mass was obtained, thus hindering processing. Similar trends have been reported for TSDs based on gelucire [27]. For hydrophilic carriers, drug solubility showed an increasing trend with a higher concentration of carrier, which may have been due to the improved wetting and solubilizing nature of surfactants [22]. However, when the weight ratio of polymers was increased beyond 40%, a turbid solution was formed. Furthermore, the solution became viscous due to the gelling properties of these carriers. Therefore, these hydrophilic carriers were used up to 40%.

**Figure 1:**
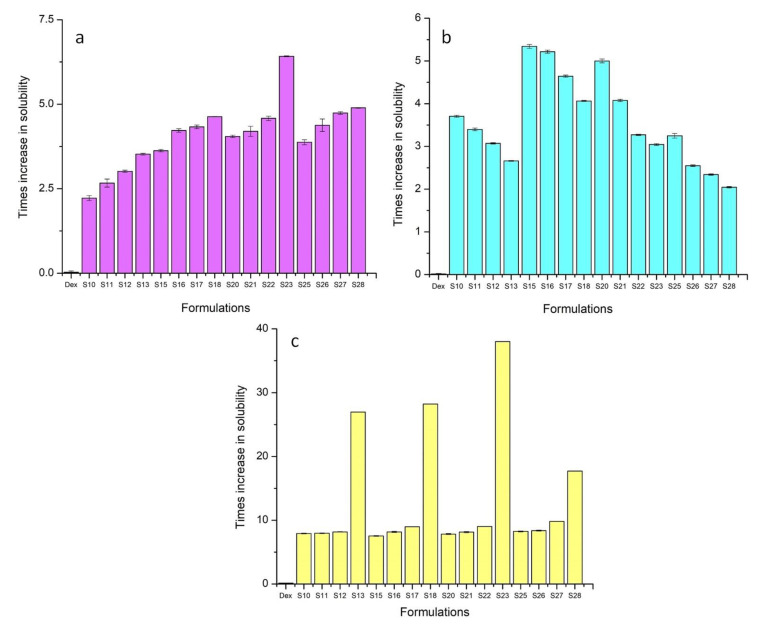
Solubility of TSD in (**a**) distilled water, (**b**) pH 1.2, and (**c**) pH 6.8. The error bar designates the standard deviation (n = 3).

All ternary carriers were able to enhance the solubility of Dex in distilled water and the buffer solution of pH 6.8 (a & c). The TSDs containing 40% Gelucire 48/16^®^ (S18) and Poloxamer 188^®^ (S23) showed the greatest increase in solubility at pH 6.8. For these formulations, the improvement in solubility was 28.23 times and 38.03 times as compared to pure drug (Figure 1). This was due to the solubilizing, wetting, and surface active nature of Gelucire 48/16^®^ and Poloxamer 188^®^ [32]. At pH 1.2, BSDs slightly increased the solubility, whereas a decreasing trend was observed from S10 to S13, S15 to S18, S20 to S23, and S25 to S28 (b). This decrease in solubility was due to the use of a higher weight ratio of ternary carriers, which provided a coating for the slow release of drug [33]. The syloid coating also contributes to the gastroprotective effect of ternary carriers [27]. The drug content of TSDs ranged from 47% to 97%. The drug content increased linearly upon increasing the weight ratio of all carriers except Soluplus^®^ (Table 5). The decreasing trend for Soluplus^®^ may have been due to the multiple hydrogen bonds between the hydroxyl groups of Soluplus^®^ with Syloid 244FP^®^ and the drug [34]. The practical yield was increased from S10 to S13, S15 to S18, and S20 to S23 due to the abundant oil adsorption capability and higher surface area of Syloid 244FP^®^ [35]. This displays the efficiency of the method employed for the development of TSDs. The practical yield decreased from S25 to S28 due to stickiness and the difficulty to scrape the formulations from the glass mortar with an increasing amount of Soluplus^®^. S18 and S23 were selected as the optimum formulations for further testing owing to the better solubility enhancement, drug content, and practical yield.

### 3.2. FTIR Analysis

**Figure 2:**
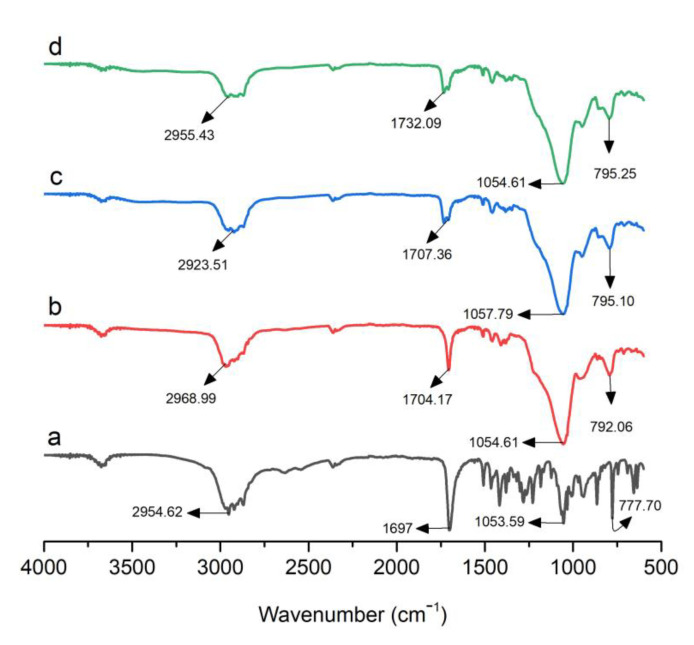
FTIR spectra of (**a**) Dex, (**b**) S1, (**c**) S18, and (**d**) S23.

The infrared spectra of pure Dex and optimized formulations, i.e., S1, S18, and S23, are presented in Figure 2. Pure Dex exhibited typical absorption peaks at 2954.62, 1697, 1053.59, and 777.70 cm^−1^ denoting C–H stretching, C=O stretching, O–H bending, and C–H bending, respectively (Figure 2). Syloid 244FP^®^ showed a strong absorption peak at 1072.35 cm^−1^ due to siloxane (Si–O–Si) stretching vibration. According to the literature, Gelucire 48/16^®^ showed peaks at 2884.30 cm^−1^ due to C–H stretching. The band at 1102.64 cm^−1^ corresponded to C–O stretching. Poloxamer 188^®^ exhibited sharp peaks at 2879.52, 1341.51, and 1097.59 cm^−1^ due to C–H stretching, in plane O–H bending, and C–O stretching, respectively. In the case of S1, S18, and S23, the absorption peaks of Dex were shifted from 1697 to 1704.17, 1732.16, and 1732.09 cm^−1^. This is possibly due to the weak interaction of Dex with the mesoporous surface, which is reversible in nature, as mentioned in the literature [27,36,37].

The FTIR spectra did not display any significant changes in the peaks of Dex in optimized formulations, suggesting a lack of incompatibility between the drug and carriers. Furthermore, hydrogen bonding between Dex and carrier molecules in the optimized formulations restricted drug recrystallization [38].

### 3.3. Powder X-ray Diffraction Analysis

**Figure 3:**
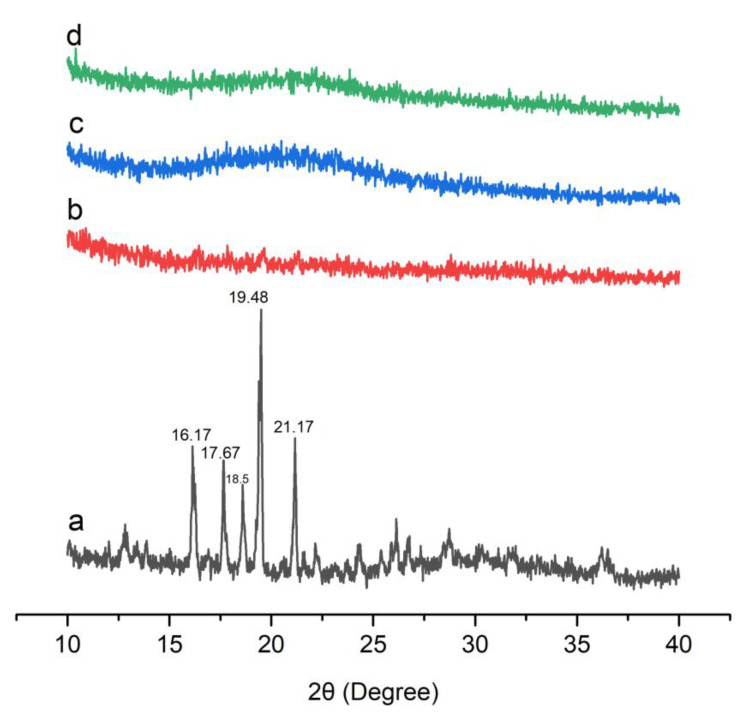
XRD graphs of (**a**) Dex, (**b**) S1, (**c**) S18, and (**d**) S23.

X-ray diffraction was used to examine the physical state of Dex and optimized formulations as presented in Figure 3. The pure Dex showed its diffraction peaks at 16.17°, 17.67°, 18.58°, 19.48°, and 21.17°. There were also a few dull peaks after 21.17°. Similar peaks were reported in the literature, indicating the crystalline nature of Dex [39].

Characteristic peaks of the drug were absent in the diffractogram of optimized formulations, suggesting entrapment of Dex in the silica pores in amorphous form [40]. The optimized formulations also showed an amorphous nature, which are further supported by the DSC results. Thus, the XRD results indicated the development of amorphous BSD and TSD formulations of Dex. Such amorphous formulations are widely employed for solubility and bioavailability enhancement of poorly water-soluble drugs.

### 3.4. SEM Analysis

**Figure 4:**
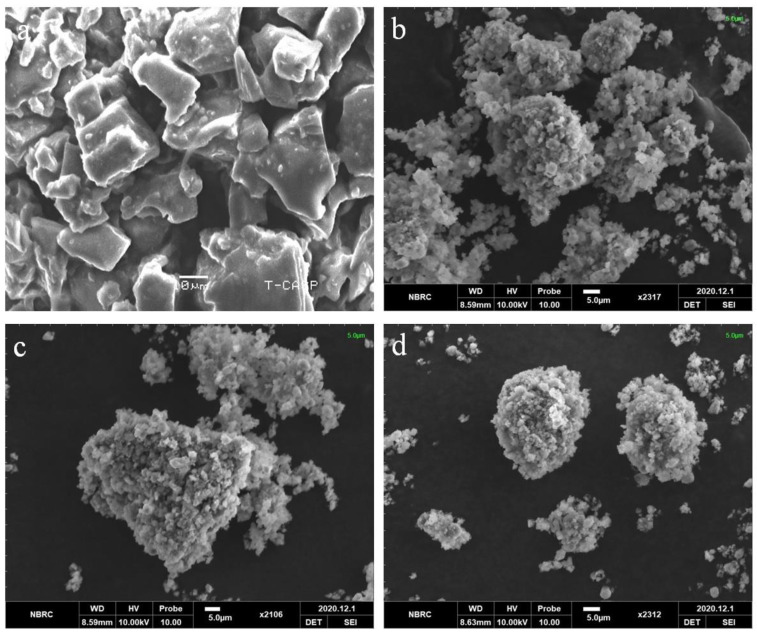
SEM images of (**a**) Dex, (**b**) S1, (**c**) S18, and (**d**) S23.

The surface morphology of pure Dex and optimized formulations were examined using SEM. The Dex image showed rectangular crystals with a smooth surface (Figure 4a), consistent with previous studies [41]. BSD and TSD appeared in aggregated clusters, which might be due to the high surface free energy of carriers [42]. No drug crystals were observed in the optimized formulations, indicating entrapment of the drug within the pores of silica in amorphous form and further confirming the XRD findings. The literature supports our findings, whereby drug confinement to silica pores prevents recrystallization of the drug, resulting in powdered dispersions. This helps to improve the solubility of the drug [27,43,44].

### 3.5. Thermal Analysis

#### 3.5.1. Thermogravimetric Analysis (TGA)

Using TGA, the responses of the pure drug and optimized formulations were assessed to check the weight loss over a span of time with a gradual increase in the temperature.

**Figure 5:**
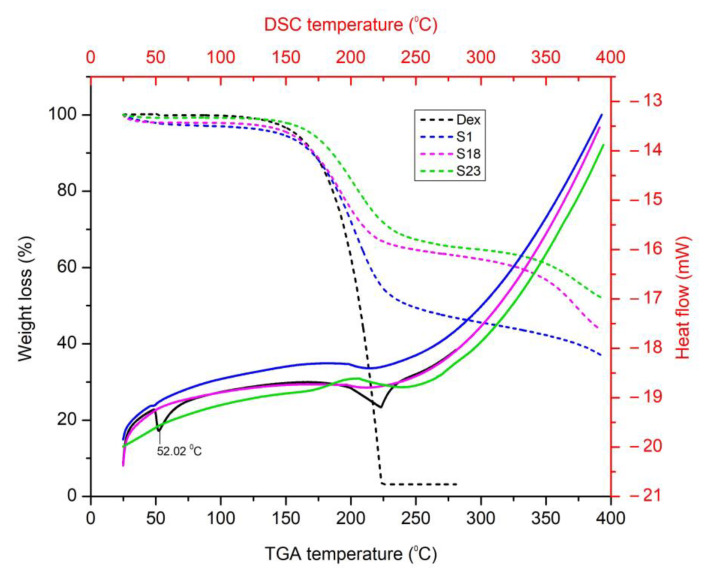
TGA and DSC graphs of Dex, S1, S18, and S23.

Two major phases of degradation were observed in the pure drug thermogram. The initial stage began from 84.87 °C and lasted up to 154.45 °C, which resulted in 4.17% weight loss (Figure 5). This initial weight loss can be attributed to the dehydration of water [45]. The second stage, ranging from 154.45 °C to 225.37 °C, resulted in 92.63% weight loss. This may have been due to degradation of drug molecules [20]. The TGA graphs of the optimized formulations revealed weight loss upon heating in three stages. Both BSD and TSD optimized formulations displayed a slow weight loss as compared to Dex. However, TSDs displayed comparatively slower degradation when compared to BSDs. This indicated that all samples of SDs were stable in the tested temperature range [22].

#### 3.5.2. Differential Scanning Calorimetry (DSC)

The DSC plots of pure Dex and optimized formulations are shown in Figure 5. The DSC graph can provide a reasonable indication of the purity, stability, and physical condition of the guest molecule within the optimized formulations. The DSC of pure Dex displayed an endothermic peak at 52.02 °C (Figure 5), corresponding to the melting point of the drug [41]. The DSC thermograms of S1, S18, and S23 did not display any melting peak of the drug at 52.02 °C. This indicates a uniform distribution of the drug in the carriers and the transformation of Dex to an amorphous state. Similar results were reported by Balakrishnan et al., who prepared an Aerosil^®^ 200-based solid SEDDS of Dex [39], as well as for the hydroxyl propyl beta cyclodextrin (HPβCD)- and poloxamer (188/407)-based inclusion complexes of Dex [41].

### 3.6. In Vitro Dissolution

**Figure 6:**
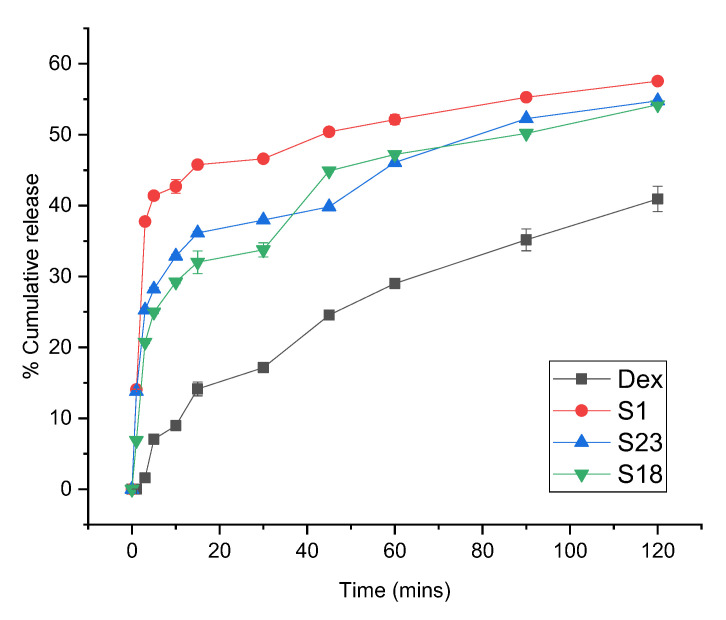
Dissolution profile of Dex and optimized formulations at pH 1.2. The error bar designates the standard deviation (n = 3).

**Figure 7:**
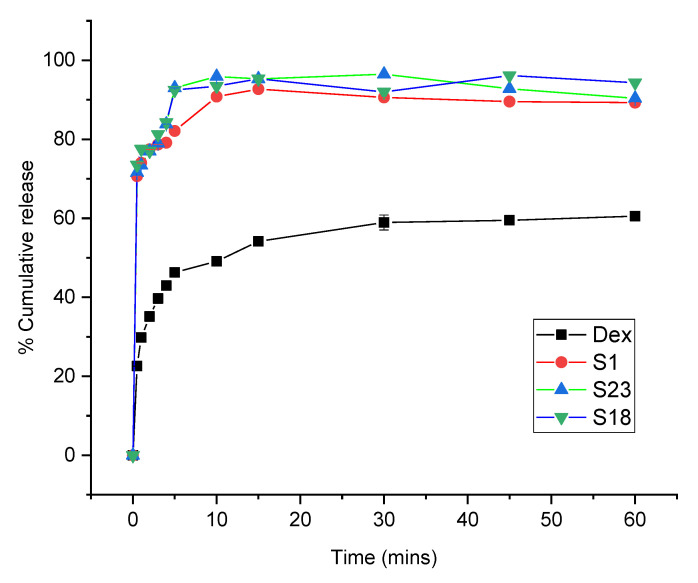
Dissolution profile of Dex and optimized formulations at pH 6.8. The error bar designates the standard deviation (n = 3).

The release profiles of Dex from the optimized formulations were conducted at pH 1.2 and 6.8 to simulate gastric and intestinal environments, respectively. Drug dissolution relies on the physicochemical properties of the drug particles. The screened formulations exhibited improved release behavior relative to pure Dex at both pH levels, as shown in Figure 6 and Figure 7.

Only 40.93% of pure Dex was dissolved in the acidic medium over the period of 2 h, whereas 1.41-fold (57.55%), 1.32-fold (54.22%), and 1.34-fold (54.77%) increases in the release profiles of S1, S18, and S23 were observed as compared to pure drug after 2 h, respectively (Figure 6). This dissolution enhancement with S1 was attributed to confinement of the drug in an amorphous form within the pores of Syloid and a weak interaction between the drug and the surface groups of silica. Previous work also demonstrated an enhancement of ibuprofen dissolution using Syloid 244FP^®^ [46]. In comparison, the dissolution profile of TSDs (S18 and S23) showed a slight decline in drug release as compared to BSD (S1). This highlights the protective role of ternary carriers in reducing the gastric-irritating effect of Dex in comparison to S1. These hydrophilic carriers can retard the release of the drug through the development of hydrogen bonding [47].

At intestinal pH, the dissolution rate was higher in comparison with pH 1.2. Dex is an acidic drug with pH-dependent solubility and release [48]. The pure drug showed burst release during the first 5 min (46.32%), followed by gradual release up to 1 h (60.53%). S1, S18, and S23 displayed 1.77-fold (82.09%), 1.99-fold (92.47%), and 2.01-fold (93.02%) increases in the dissolution rate during first 5 min as compared to pure drug (Figure 7). This quick release from S1 was attributed to the porous nature of Syloid 244FP^®^, which prevented crystallization of the drug and enabled the dissolution medium to rapidly diffuse into the silica pores, resulting in immediate dislocation of the drug [46]. As shown by the FTIR results, the weak interaction between Dex and silica also contributed to an enhancement of Dex dissolution. Overall, a higher release pattern was attained with S18 (96.14%) and S23 (96.50%) during the first 10 min. S18 showed an improvement in the dissolution rate by inhibiting the drug crystal growth and micellar solubilizing action of Gelucires^®^ [27,49]. The superior drug release profile of S23 was due to the surface-active nature of Poloxamer 188^®^. There was a slight decline in drug release from S1, S18, and S23 after 15 min owing to supersaturation. This was based on the fact that the amorphous form was at a higher energy level; thus, precipitation could lead to a small decrease in the release pattern [50].

### 3.7. Micromeritic Properties

The micromeritic evaluation showed that BSDs and TSDs displayed better flow characteristics when compared to the pure drug (Table 6).

The values of Carr’s index (33.85%), Hausner’s ratio (1.51), and angle of repose (56°) for Dex highlighted its poor flow attributes, whereas the flowability of the optimized formulations was within the official limits (Table 6). Syloid 244FP^®^ is a multipurpose excipient that acts as a glidant and improves the flow properties. S18 showed a further improvement in flow properties when compared with S1, which was attributed to the presence of the lipid portion in Gelucire 48/16^®^ that provided lubrication for ease of flow [27,51].

### 3.8. Assessment of Gastro Protective Effect In Vivo

#### 3.8.1. Macroscopic Scoring

**Figure 8:**
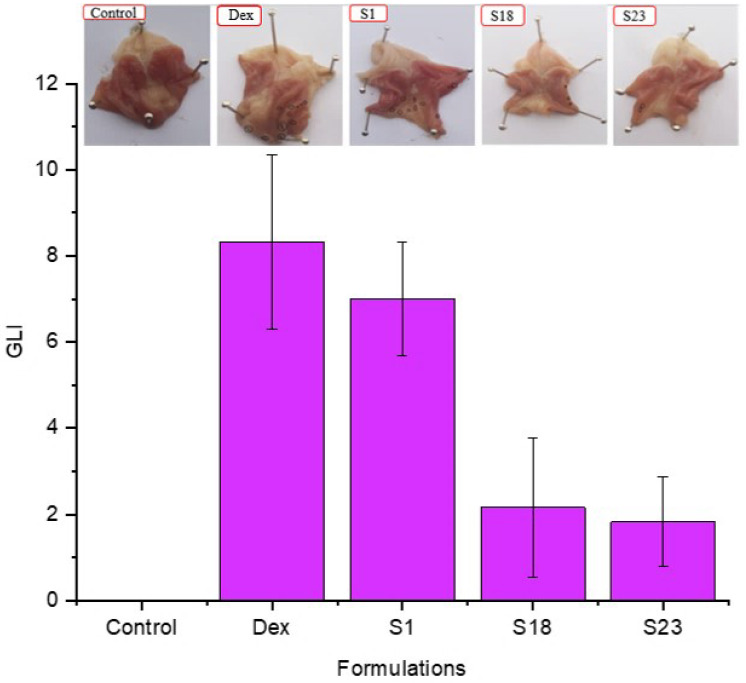
Stomach mucosa of rats and gastric lesions scores. The error bar designates the standard deviation (n ꞊ 3).

Macroscopic lesions in the stomach mucosa of rats from each group are shown in Figure 8. The trend of average GLI was in the order of pure Dex (8.33 ± 2.02) > S1 (7 ± 1.32) > S18 (2.17 ± 1.61) > S23 (1.83 ± 1.04) > control (0).

According to the GLI, pure drug and S1 displayed a higher lesion score as compared to S18 and S23. Dex belongs to a group of analgesic drugs that cause gastric irritation upon administration. S1 ulcerated the gastric mucosa as silica carriers are notorious for inducing membrane damage. This response was attributed to silanol groups of silica particles, which causes cellular damage and hemolysis through the development of hydrogen bonding with the cell membrane, as previously reported [27]. Moreover, Poloxamer 188^®^ (S23) and Gelucire 48/16^®^ (S18) prevented the damage of the gastric mucosa by reducing the lysis of the cell membrane [27,52]. This was achieved via a reduction in the direct interaction between the carboxylic acid group of Dex and silanol groups with the gastric mucosa. A previous study demonstrated the neuroprotective role of Poloxamer 188^®^ [53]. Our results clearly demonstrate that the side-effects of silica and drug could be minimized using TSD-based formulations.

#### 3.8.2. Histopathological Analysis

**Figure 9:**
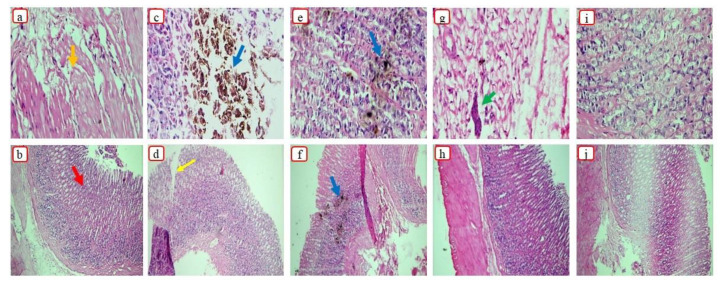
Histological presentation of rat’s stomach in the (**a**,**b**) control, (**c**,**d**) Dex, (**e**,**f**) S1, (**g**,**h**) S18, and (**i**,**j**) S23 groups. Here, the orange arrow indicates the mucosa, the red arrow indicates gastric pits, the blue arrow indicates necrotic lesions, the yellow arrow indicates the damaged epithelium, and the green arrow indicates fibrin deposition.

Histological images of the stomach are shown in Figure 9. The rat’s stomach from the control group displayed intact glands, a simple columnar epithelium, and a normal mucosa [54].

The group treated with pure Dex displayed necrotic lesions in the mucosa, as well as a damaged epithelium, due to the interaction between the mucosa and carboxylic acid groups of the drug. Dex is responsible for inhibiting COX-2. Prostaglandins generated by COX-2 contribute to the improvement of vessel constriction, permeability, fever, and pain linked with inflammation. Therefore, the administration of Dex can result in bleeding and damage to epithelium [55]. Histopathological photographs of the rat’s stomach treated with S1 displayed epithelial damage, as well as necrotic lesions, as silica can disrupt the cellular membrane [56]. Therefore, both Syloid 244FP^®^ and pure drug were responsible for damaging the mucosal membrane. The group treated with S18 showed some fibrin depositions. Gelucire 48/16^®^ can help to protect the mucosal membrane by preventing direct contact of the drug with the gastric membrane, as previously demonstrated in many studies where lipid-based formulations protected against ethanol-induced ulceration [57]. Furthermore, a reduction in GLI was reported when aspirin was encapsulated in lipid-based carriers [58]. Micrographs of the rat’s stomach treated with S23 showed a normal mucosa, with intact muscularis mucosa and blood vessels. As stated earlier, Poloxamer 188^®^ has the potential to protect the gastric mucosa by preventing breakdown of the gastric membrane [59]. Furthermore, Poloxamer 188^®^ is also helpful in enhancing cellular proliferation and tissue repair by inducing the synthesis of epidermal growth factor [60].

### 3.9. Evaluation of Anti-Inflammatory Activity

Pure drug and optimized formulations were assessed for the percentage reduction in inflammation, and the results were compared with the control group. The data were analyzed using two-way ANOVA through GraphPad Prism, where the level of significance was considered as slightly significant (* *p <* 0.05), moderately significant (** *p <* 0.01), and highly significant (*** *p <* 0.001) for all treatment groups as compared to the control group. All treatment groups showed a highly significant (*p <* 0.001) reduction in inflammation when compared for six consecutive hours.

**Figure 10:**
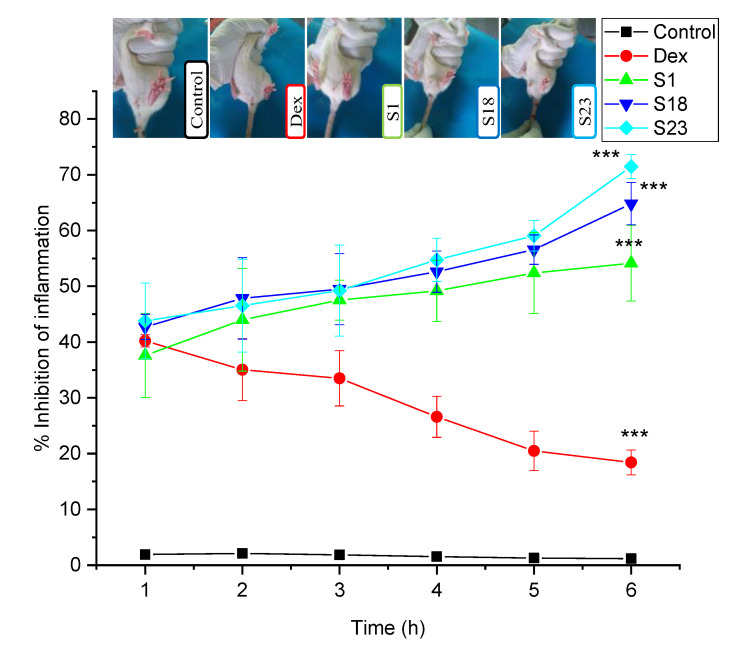
Photographs of rat paw edema after 6 h and percentage inhibition of carrageenan-induced paw edema in rats. The error bar designates the standard deviation (n = 3). Here, the control refers to the diseased group, which did not receive any treatment. Here, level of significance was slightly significant, moderately significant and highly significant (*** *p <* 0.001) for all treatment groups when compared with control group.

The anti-inflammatory activity of pure Dex declined with time, whereas the optimized formulation showed an increase in anti-inflammatory activity with time. This trend was possibly due to the lower bioavailability of pure drug in comparison with the optimized formulations (Figure 10). Similar results were observed in a previous study, where the amide-based prodrug of pure Dex was formulated using amino acids [26]. In another study, the authors developed biphasic tablets of lornoxicam, featuring an immediate release layer comprising an SD of lornoxicam and a controlled release layer comprising microspheres of the drug. The anti-inflammatory activity showed 48% inhibition of inflammation during the first 30 min, which increased to 88.63% after 4 h. In this case, the immediate layer provided the initial inhibition, while the sustained layer maintained the level of drug in the blood for prolonged inhibition [61].

The percentage inhibition of swelling was in the order of S23 (71.47 ± 2.16) > S18 (64.8 ± 3.79) > S1 (54.14 ± 6.78) > pure drug (18.43 ± 2.21) > control (1.18 ± 0.64) after 6 h. The results of TSDs were superior when compared with BSDs, demonstrating that the combination of silica, gastroprotective polymer, and lipids provided controlled release and enhanced the bioavailability of Dex.

#### 3.9.1. Measurement of Serum IL-6 and TNF Alpha

**Figure 11:**
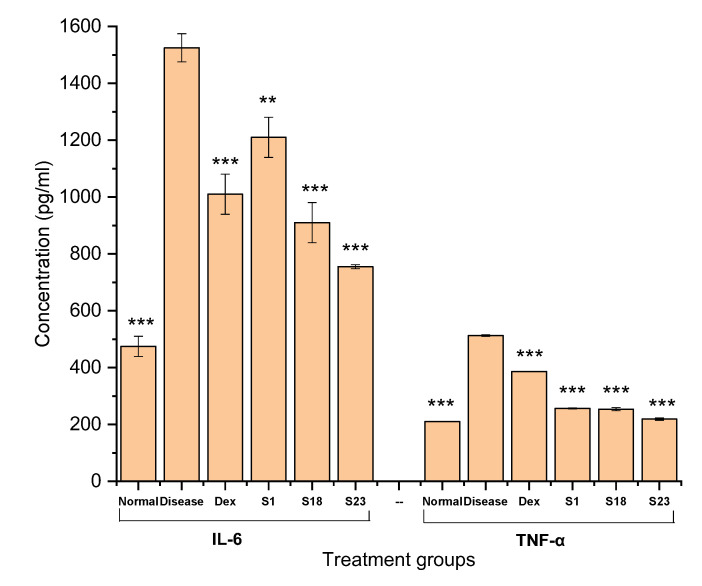
Serum IL-6 and TNF-α levels. The error bar designates the standard deviation (n ꞊ 3, *p* < 0.05). The data are represented as the mean ± S.D. The comparison with the diseased control group is denoted by ** (moderately significant) and *** (highly significant) at *p* < 0.01 and *p* < 0.001, respectively. Here, normal refers to the group, which did not receive carrageenan and treatment.

Inflammatory cytokines (IL-1, IL-6, and TNF- α) play a significant role in disease pathology. Therefore, measurement of these mediators in the serum is important. In the current study, IL-6 and TNF alpha were quantified using ELISA. ELISA revealed increased levels of IL-6 in the diseased group, indicating an induction of inflammation (1525 ± 49.50 pg/mL), whereas the control group showed the lowest level (Figure 11).

The S1-treated group presented a high IL-6 level (1210 ± 70.71 pg/mL) in comparison with the Dex, S18, and S23 groups (Figure 11). This behavior was due to the toxic effects of silica [62]. On the other hand, the S23 group showed significantly low levels of IL-6 when compared with the diseased group, representing the efficacy of the TSD formulation in inhibiting inflammation.

TNF alpha levels were recorded in the order of diseased group (513 ± 2.83 pg/mL) > Dex (386.5 ± 0.71 pg/mL) > S1 (256.5 ± 2.12 pg/mL) > S18 (254 ± 5.66 pg/mL) > S23 (219 ± 4.24 pg/mL) > normal group (210.5 ± 0.71 pg/mL). S23 displayed substantially low levels of TNF alpha in comparison to the diseased group. It was also reported in the literature that Poloxamer 188^®^ lowers the levels of inflammatory cytokines (IL-1 and TNF-α) that play a significant role in disease pathology [63]. Curcumin is a powerful anti-inflammatory agent. However, its low aqueous solubility, rapid metabolism, stability issues, and low oral bioavailability affect its efficacy. An amorphous solid dispersion of curcumin was developed, which enhanced bioavailability 11-fold and significantly reduced cytokine levels when compared to pure curcumin [64].

#### 3.9.2. Histopathology of Rat Paw

**Figure 12:**
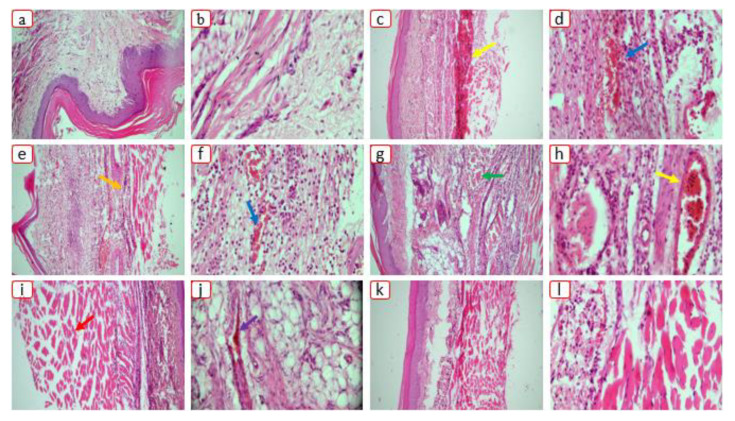
Histological appearance of the rat paw edema in (**a**,**b**) normal group, (**c**,**d**) diseased group, (**e**,**f**) Dex group, (**g**,**h**) S1group, (**i**,**j**) S18 group, and (**k**,**l**) S23 group. Here, the yellow arrow indicates cellular infiltration, blue arrow indicates inflammation, orange arrow indicates hyperplasia, green arrow indicates giant cells, red arrow indicates blood vessel, and purple arrow indicates bone erosion.

Cellular infiltrations were seen in the paw tissue in the untreated control group (Figure 12). On the other hand, rats treated with pure Dex and optimized formulations showed a decline in inflammatory responses.

## 4. Conclusions

In the current study, BSDs and TSDs of Dex were successfully formulated to address its issues related to solubility and bioavailability. Initially, nine BSDs were developed with three different Syloid carriers. Among them, S1 containing a 1:1 weight ratio of Dex and Syloid 244FP^®^ was selected for the development of TSDs owing to better drug entrapment (97%) and enhanced solubility of the drug when compared to pure Dex. Subsequently, TSDs were developed using two lipophilic carriers (Gelucire 44/14^®^ and Gelucire 48/16^®^) and two hydrophilic carriers (Poloxamer 188^®^ and Soluplus^®^). S18 (Gelucire 48/16^®^) and S23 (Poloxamer 188^®^) were chosen for further analysis owing to improvements in the solubility, drug content, and practical yield. FTIR analysis did not reveal any significant interaction with the carrier, while XRD analysis indicated the presence of drug in amorphous form. TSDs showed better flow properties when compared with BSDs and pure drug. Furthermore, TSDs showed a better release of drug when compared to pure Dex. TSDs exhibited better gastroprotective and anti-inflammatory effects when compared with Dex and BSDs during in vivo testing. These findings successfully demonstrate the suitability of Gelucire 48/16^®^ and Poloxamer 188^®^-based TSDs for tackling the bioavailability issues and minimizing the gastric irritation of Dex. In the future, further testing of S23 in humans is required to confirm its suitability.

## Figures and Tables

**Table 1:** Composition of BSDs.

Sr. No.	Sample No.	Grade of Silica	Drug: Silica (*w*/*w*)
1	S1	Syloid 244FP^®^	1:1
2	S2	Syloid 244FP^®^	1:2
3	S3	Syloid 244FP^®^	1:4
4	S4	Syloid AL1FP^®^	1:1
5	S5	Syloid AL1FP^®^	1:2
6	S6	Syloid AL1FP^®^	1:4
7	S7	Syloid XDP3150^®^	1:1
8	S8	Syloid XDP3150^®^	1:2
9	S9	Syloid XDP3150^®^	1:4

**Table 2:** Composition of TSDs.

Sr. No.	Sample No.	Grade of Silica	Ratio of Drug to Silica (*w*/*w*)	Ternary Carrier	Percentage of Ternary Carrier (*w*/*w*)
1	S10, S11, S12, S13, and S14	Syloid 244FP^®^	1:1	Gelucire 44/14^®^	5%, 10%, 20%, 40%, 80%
2	S15, S16, S17, S18, and S19	Syloid 244FP^®^	1:1	Gelucire 48/16^®^	5%, 10%, 20%, 40%, 80%
3	S20, S21, S22, S23, and S24	Syloid 244FP^®^	1:1	Poloxamer 188^®^	5%, 10%, 20%, 40%, 80%
4	S25, S26, S27, S28, and S29	Syloid 244FP^®^	1:1	Soluplus^®^	5%, 10%, 20%, 40%, 80%

**Table 3:** Properties of mesoporous silica.

Silica	Particle Size (µm)	Surface Area (m^2^/g)	Pore Diameter (nm)	Pore Volume (cm^3^/g)
Syloid 244FP^®^	2.5–3.7	379	17	1.6
Syloid ALIFP^®^	6.5–8.1	605	26	0.3
Syloid XDP3150^®^	110	320	200	1.7

**Table 4:** Solubility, percentage drug, and percentage yield of binary formulations.

Sr. No.	Formulation	Solubility (mg/mL)			% Drug	% Yield
		Distilled H₂O	pH 1.2	pH 6.8		
1	Dex	0.029 ± 0.04	0.016 ± 0.03	0.13 ± 0.01	-	-
2	S1	0.18 ± 0.09	0.028 ± 0.014	3.12 ± 0.06	97	91
3	S2	0.22 ± 0.15	0.036 ± 0.003	2.95 ± 0.02	93	93
4	S3	0.24 ± 0.18	0.059 ± 0.008	2.91 ± 0.04	92	96
5	S4	0.06 ± 0.002	0.002 ± 0.009	0.59 ± 0.006	66	83
6	S5	0.09 ± 0.004	0.005 ± 0.005	0.63 ± 0.002	81	84
7	S6	0.10 ± 0.001	0.006 ± 0.002	0.72 ± 0.001	86	87
8	S7	0.05 ± 0.002	0.003 ± 0.01	0.66 ± 0.03	65	94
9	S8	0.072 ± 0.003	0.007 ± 0.03	0.60 ± 0.01	59	95
10	S9	0.073 ± 0.001	0.008 ± 0.01	0.58 ± 0.02	56	96

**Table 5:** Solubility, percentage drug content, and percentage yield of ternary formulations.

Sr. No.	Formulation	Solubility Studies (mg/mL)			Drug Content	% Yield
		Distilled H₂O	pH 1.2	pH 6.8		
1	S10	0.064 ± 0.07	0.062 ± 0.02	1.031 ± 0.07	70%	94.28%
2	S11	0.076 ± 0.12	0.057 ± 0.03	1.036 ± 0.04	91%	94.54%
3	S12	0.087 ± 0.03	0.052 ± 0.01	1.063 ± 0.04	94%	95.83%
4	S13	0.101 ± 0.03	0.045 ± 0.01	3.51 ± 0.02	96%	96.42%
5	S15	0.104 ± 0.03	0.090 ± 0.04	0.98 ± 0.07	60%	89.52%
6	S16	0.121 ± 0.05	0.088 ± 0.03	1.061 ± 0.08	73%	90.00%
7	S17	0.125 ± 0.05	0.079 ± 0.02	1.168 ± 0.02	82%	96.66%
8	S18	0.133 ± 0.01	0.068 ± 0.01	3.67 ± 0.01	97%	97.86%
9	S20	0.117 ± 0.04	0.084 ± 0.04	1.019 ± 0.07	58%	91.43%
10	S21	0.121 ± 0.15	0.069 ± 0.02	1.058 ± 0.11	76%	92.73%
11	S22	0.132 ± 0.07	0.055 ± 0.01	1.174 ± 0.02	80%	94.16%
12	S23	0.185 ± 0.02	0.052 ± 0.02	4.943 ± 0.01	91%	95.71%
13	S25	0.112 ± 0.07	0.055 ± 0.05	1.074 ± 0.07	65%	87.62%
14	S26	0.126 ± 0.18	0.043 ± 0.01	1.090 ± 0.08	62%	87.27%
15	S27	0.137 ± 0.04	0.039 ± 0.01	1.277 ± 0.01	56%	84.16%
16	S28	0.141 ± 0.01	0.035 ± 0.02	2.303 ± 0.01	47%	82.86%

**Table 6:** Flow properties of optimized formulation powders.

Sr. No.	Samples	Bulk Density gm/mL	Tapped Density gm/mL	Carr’s Index%	Hausner’s Ratio	Angle of Repose (θ)	Remarks
1	Dex	0.170	0.257	33.85	1.51	56	Very poor
2	S1	0.234	0.270	13.33	1.15	31	Good
3	S18	0.239	0.266	10.15	1.11	26	Excellent
4	S23	0.234	0.259	9.65	1.10	23	Excellent

## Data Availability

Most of the data for the current study are presented in the article. However, the raw or processed data required to reproduce these findings cannot be shared at this time due to technical or time limitations.
